# The hypothalamic-neurohypophyseal system in preeclampsia: a systematic review with a subgroup meta-analysis of copeptin levels worldwide

**DOI:** 10.3389/fendo.2026.1796685

**Published:** 2026-06-10

**Authors:** Gustavo M. Moreira, Roger Rodríguez-Guzmán, Naima J. S. Marciano, Isabely G. Silva, Anja Babic, Elias J. M. Seif, Camila Nascimento, Nelson Sass, André S. Mecawi

**Affiliations:** 1 Laboratory of Molecular Neuroendocrinology, Department of Biophysics, Federal University of São Paulo, São Paulo, Brazil; 2 School of Medicine, Dentistry and Biomedical Sciences, Queen’s University Belfast, Belfast, United Kingdom; 3 Department of Biochemistry, Federal University of São Paulo, São Paulo, Brazil; 4 Department of Obstetrics, Federal University of São Paulo, São Paulo, Brazil

**Keywords:** arginine vasopressin, copeptin, meta-analysis, preeclampsia, surrogate

## Abstract

Literature has reported elevated concentrations of copeptin (CPP), a marker of arginine vasopressin (AVP) secretion, preceding the clinical features of the life-threatening condition, preeclampsia (PE). Those findings pointed to a possible role for the hypothalamic-neurohypophyseal system (HNS) in its pathophysiology. This systematic review and meta-analysis reports CPP levels in women with PE, exploring the AVP system not as a primary driver of the disease, but as a dynamic physiological feature associated with its clinical progression. The search terms used were “preeclampsia-AND-vasopressin-OR-copeptin-OR-oxytocin” in five databases: Google Scholar, Embase, Scopus, PubMed, and Semantic Scholar. Study quality was assessed using the Newcastle-Ottawa Scale. A Gaussian Mixture Model (GMM) algorithm was applied to blind predict the preeclamptic or healthy normotensive pregnancies by the CPP concentration, after performing a Kernel Principal Component Analysis (kPCA). Thirty-nine registries were selected, of which twenty were observational studies documenting CPP concentrations across all three trimesters, encompassing 1025 cases in 2446 pregnancies. Observational and experimental studies have associated the development of PE with circulating CPP levels and pharmacological manipulations involving AVP. Such an association was not found for oxytocin. Continental comparisons reveal a potential gene-environment link, showing a greater effect of higher concentrations of CPP on preeclamptic cases in Asian and African people. Furthermore, gestational hypertension has been associated with single nucleotide polymorphisms (SNPs) in the *AVP* and *ERAP2* genes. Notably, *ERAP2* encodes an endopeptidase that cleaves and inactivates AVP. It is essential to improve the early detection of this condition by understanding how genetic and geographic factors influence PE causes.

**Systematic review registration:**

https://www.crd.york.ac.uk/prospero/, identifier CRD420251111755.

## Graphical Abstract

**Figure:**
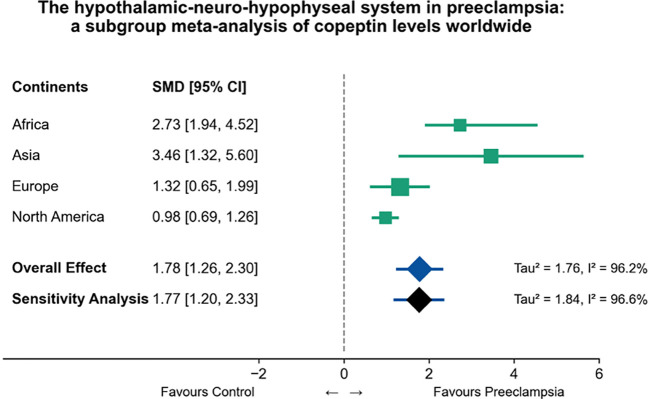

## Highlights

- This systematic review and meta-analysis confirms that elevated CPP, a surrogate for AVP, is a consistent marker for PE, unlike oxytocin. Analysis of 39 studies revealed that genetic factors influence this association, with even higher CPP levels observed in Asian and African cases. These findings underscore the role of the HNS in the pathophysiology of PE.

## 1 Introduction

Pregnancy brings about extreme physiological changes due to the complex interaction established at the maternal-fetal interface (1). Preeclampsia (PE) is a life-threatening maternal condition specific to pregnancy, characterised by the appearance of high blood pressure after the 20th week of pregnancy, which may or may not be accompanied by proteinuria and/or organ dysfunction (Shir-Jing 2, 3). Besides its considerable risk of morbidity and mortality, it can occur in up to 8% of pregnancies in some locations, such as Brazil’s lowest-income regions (4, 5).

Labour is the only definitive treatment that successfully halts progression (ACOG Practice Bulletin Summary of 6). If not diagnosed and treated early, PE can lead to the development of more serious conditions, such as eclampsia, haemorrhagic stroke, HELLP syndrome (haemolysis, elevated liver enzymes, and low platelet count), renal failure and/or acute pulmonary oedema (7). During early pregnancy, the hypothalamic-neurohypophyseal system (HNS) undergoes significant modifications, including an increase in arginine vasopressin (AVP) secretion to promote water retention and expansion of the extracellular fluid volume, ensuring adequate placental perfusion (8, 9).

The magnocellular neuron cells located in the paraventricular and supraoptic nuclei of the hypothalamus are responsible for the synthesis and storage of AVP, Copeptin (CPP), and oxytocin, which are subsequently released into the bloodstream via the neurohypophysis (10, 11). Upon entering the bloodstream, AVP acts on the renal collecting tubules, promoting the insertion of aquaporin 2 channels to improve water reabsorption. Simultaneously, it induces vasoconstriction in the smooth muscle cells of blood vessels, elevating blood pressure (11, 12).

The action of the AVP comes to an end when it is metabolized by circulating peptidases, including leucyl/cystinyl aminopeptidase (LNPEP), insulin-regulated aminopeptidase (IRAP), and endoplasmic reticulum aminopeptidase 2 (ERAP2) (13, 14). Nevertheless, since 1990, a hypothesis by Krege and Katz suggested that the abnormal activity of vasopressinases contributes to hypertension in the placenta. Furthermore, studies across various populations have demonstrated an association between PE and single nucleotide polymorphisms (SNPs) located within the *AVP* and *ERAP2* genes. However, utilizing these genetic markers or their associated biomarkers to predict PE often yields low diagnostic sensitivity, particularly in low-risk pregnancies. This suggests that their predictive value might be clinically relevant only in specific scenarios (15, 16).

The onset of PE is generally understood to originate from the incomplete transformation of the endometrial spiral arteries during the first months of gestation, which subsequently leads to placental hypoperfusion (17). Research indicates that early-onset (EOPE) and late-onset (LOPE) forms, occurring before or after the 34th week, respectively, possess distinct molecular profiles and pathophysiological drivers (18, 19). Furthermore, chronic exogenous AVP administration in pregnant mice has been shown to induce PE-like symptoms, including renal and placental damage and cytokine imbalances (20–22). These effects, particularly regarding T-helper cell dysfunction, suggest that AVP signaling may trigger the immunological shifts characteristic of the condition (22).

CPP is the C-terminal sequence of pre-pro-vasopressin produced by the same gene as AVP at a 1:1 ratio, making it an indirect but more stable indicator of AVP production and secretion (23, 24). CPP concentration as an early prognostic PE biomarker is a common theme in the literature. Multiple studies demonstrate that CPP levels are elevated as early as the first trimester, long before clinical onset, and that these levels correlate with the eventual severity of the disease (25, 26). The use of plasma CPP levels as an early marker for the development of PE has been the subject of patent applications (WO 2014/124392 International Application No. PCT/US2014/015627).

Besides the fact that CPP has been widely used to infer AVP secretion, its potential bioactive circulatory effects remain unclear since potential cognate receptors are still unknown (27). The collective findings suggest CPP as a key molecule for understanding PE, with implications in early detection, underlying pathophysiology, and long-term health effects (28–30). This systematic review aims to evaluate the evidence on the association between the peripheral concentration of the HNS hormones (AVP/CPP or oxytocin) and PE development. The primary objective of the meta-analysis is to quantify the overall association between circulating CPP levels and PE. The secondary objective is to explore sources of heterogeneity by conducting a subgroup meta-analysis based on geographic region and reported ethnicity or genetic factors.

## 2 Methods

### 2.1 Systematic search

The Preferred Reporting Items for Systematic Reviews and Meta-Analyses (PRISMA) standards were followed (31 32) and the protocol was registered in PROSPERO as: CRD420251111755, and can be found online at: https://dx.doi.org/10.17504/protocols.io.bp2l6zne1gqe/v1. The search was conducted between March and September-2025, using specific syntaxes for each database:

- PubMed: (preeclampsia[MeSH Terms] OR “pre-eclampsia” OR “gestational hypertension”) AND (vasopressin[MeSH Terms] OR “arginine vasopressin” OR “copeptin” OR “oxytocin”);
- Embase: (‘preeclampsia’/exp OR ‘preeclampsia’ OR ‘gestational hypertension’) AND (‘vasopressin’/exp OR ‘vasopressin’ OR ‘copeptin’ OR ‘oxytocin’);
- Scopus: TITLE-ABS-KEY((“preeclampsia” OR “pre-eclampsia”) AND (“vasopressin” OR “copeptin” OR “oxytocin”));
- Google Scholar: “preeclampsia” “vasopressin” OR “copeptin” OR “oxytocin”;
- Semantic Scholar: (preeclampsia) AND (vasopressin OR copeptin OR oxytocin).

This work aimed to respond to the following research questions: Is PE associated with AVP or CPP in the literature consulted? Is there a link between the prevalence of PE and increased levels of CPP in a geographically dependent manner? And finally, are there PE-related genetic variations in the HNS-related genes? To answer those questions, the PECO standard (population, exposure, comparator and outcome) was used to outline the search criteria and the approaches to assess the quality of each study. The population was defined as “pregnant women in any gestational trimester”, the exposure was defined as “preeclampsia”, “healthy pregnancies” was defined as the comparison group, and the outcome assessed was defined as “the imbalance of the HNS in PE”.

### 2.2 Inclusion and exclusion criteria

Original studies available that analysed the peripheral concentration of hormones such as AVP and CPP relating them to the development of PE in full text and written in English were included in this review. Eligible studies comprised case-control, cross-sectional, and longitudinal designs. Studies were also included regardless of the trimester of pregnancy (<14 weeks of gestation for the first trimester, from 14th to 27th weeks for second trimester, and as >28th weeks for the third one). There were no restrictions regarding the year of publication either. In addition, we included in the systematic review studies evaluating genomic variants known to influence the development of PE, particularly those involving genes related to AVP signalling and metabolism. These studies were selected to allow us to summarise the frequencies of relevant SNPs in a table, highlighting how geographic location may influence CPP concentrations in those cases.

For the meta-analysis, systematic or narrative reviews, meta-analyses, dissertations, thesis, undergraduate research papers, e-books, *in vitro* or animal studies, or studies not available in full text were excluded.

### 2.3 Data extraction and screening

Initial screening through titles and abstracts was according to the PECO standard to ensure all records met the predefined eligibility criteria. After identifying records across five databases, duplicates were removed, and the remaining titles and abstracts were reviewed using the Rayyan software. Any confusions regarding retrieval or study selection were resolved by consensus between the researchers.

### 2.4 Random-effects meta-analysis

A random-effects association meta-analysis with pooled estimates of the standardised mean difference (SMD) and 95% confidence intervals (CI) was employed to determine the combined mean effect based on the inverse variance. For the meta-analysis, data on peripheral CPP concentration from preeclamptic cases and healthy normotensive pregnancies worldwide reported within 20 selected records was used. All analyses were carried out by Python (‘Python Software Foundation’) employing the Der Simonian and Laird methods to build forest plots. The studies’ heterogeneity (Tau^2^) and the Inconsistency Indexes (I^2^) were also assessed. To explore potential geographic influences on CPP elevation in PE, a subgroup meta-analysis was performed, stratifying data by region of sampling.

### 2.5 Risks of bias and sensitivity analysis

The methodological quality of the evidence of all the included studies was assessed using the Newcastle-Ottawa Scale score and the Grading of Recommendations Assessment Development and Evaluation (GRADE) framework. Case-control studies were assessed considering factors such as the selection of cases, the comparability of groups and the determination of exposure. Cohort studies were assessed according to the selection process, the comparability of exposed and unexposed groups, and the evaluation of results. To assess the risk of bias, a funnel plot was used to check for significant heterogeneity and asymmetry. The sensitivity analysis was conducted by excluding the CPP values derived from severe PE samples.

### 2.6 Application of CPP concentration as a PE-risk factor to a clustering algorithm

To explore the distributive patterns of the data, a clustering algorithm was employed to examine the separation between healthy normotensive pregnancies and PE according to mean CPP concentrations extracted from the 20 included studies. The analysis was conducted in Python using the libraries: Pandas (33), NumPy (34), and Scikit-learn (35). To address inter-study variability, a mean Z-score paired-normalization technique was applied, and all values were standardized to pg/mL. Standard deviation (SD) was either used directly or estimated from interquartile ranges (SD≈IQR/1.35) to ensure a complete dataset. The analysis was weighted by the sample size of each study.

A Kernel Principal Component Analysis (kPCA) was subsequently applied to the weighted and normalized data to account for potential non-linear relationships and to facilitate visual exploration of the cohorts (36). A Gaussian Mixture Model (GMM) was then applied to the first two principal components (kPC1 and kPC2). GMM is a probabilistic, unsupervised learning method that assumes the data is composed of a mixture of Gaussian distributions (37). This approach utilizes an Expectation-Maximization algorithm (38) to iteratively calculate the probability of each data point belonging to one of two clusters without prior knowledge of the true labels.

## 3 Results

### 3.1 The HNS dysregulation in PE

A total of 39 peer-reviewed articles, published from 1985 to 2025, were incorporated into this systematic review. This evidence base offers an updated overview of the HNS-PE association; comprehensive data for each study is presented in **Supplementary Table 1**. In summary, the data implicates AVP and CPP consistently in the condition’s development, however research focusing on oxytocin’s association with PE was insufficient. The effects of AVP/CPP dysregulation were reported to extend beyond pregnancy, with persistently elevated CPP levels detected for years postpartum, which were associated with a higher long-term cardiovascular risk for the mother (39, 40). Santillan et al. (41) and Sandgren et al. (21) verified evidence of induced PE-like symptoms from animal models with the AVP infusion alone, such as hypertension and fetal growth restriction.

Shir-Jing Ho et al. (2) and Gao et al. (42) found that placental impairment was linked to local AVP signaling and epigenetic changes, specifically the hypermethylation of AVP receptor genes. Yeung et al. (26) detected elevated CPP levels as early as six weeks of gestation, prior to appearance of clinical symptoms, while Aboelmagd et al. (25) showed that there is a rise in CPP correlated with PE severity. Genetic research also identified the presence of SNPs in the *AVP* and *ERAP2* genes that influence PE risk and blood pressure regulation in certain populations. Regarding fetal effects, prenatal exposure to AVP was linked to altered AVP signaling and subsequent sex-specific neurodevelopmental changes in offspring (12).

### 3.2 Included studies in the meta-analysis

**Figure 1:**
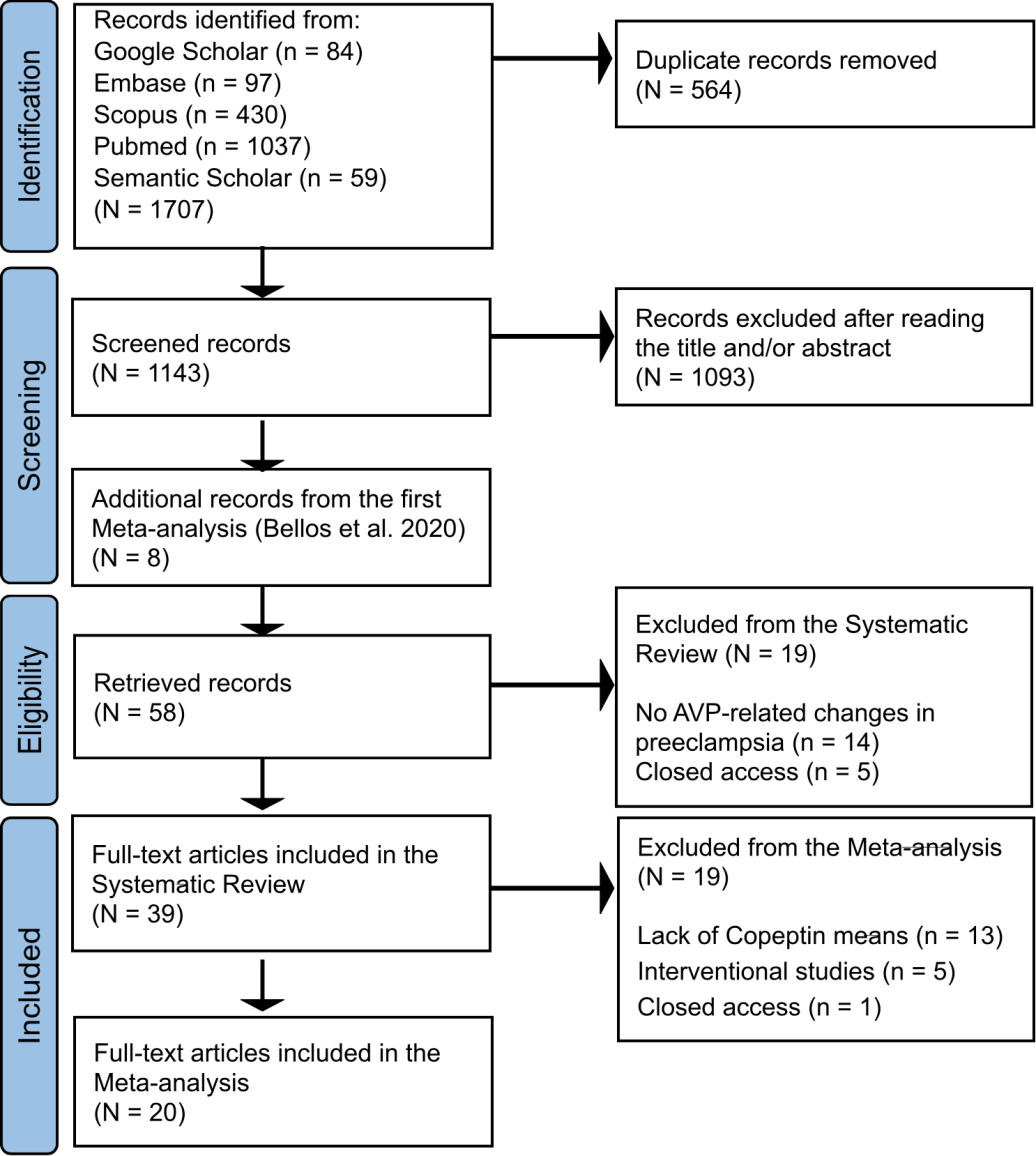
Flowchart of selected records in the meta-analysis. It outlines the steps for identifying eligible studies.

The meta-analysis comprised 20 studies, from which a total of 27 independent data sets were extracted, encompassing a total of 2446 participants, 1421 healthy normotensive pregnancies and 1025 preeclamptic. The reasons for each author’s exclusion from the meta-analysis are listed in **Supplementary Table 2**. The specific steps of the systematic search and the identification of the final database of eligible studies for the meta-analysis are illustrated in the results section (**Figure 1**).

Among the twenty studies, fourteen reported a single consolidated outcome, while the remaining six studies provided stratified results: three reported separate data for mild and severe PE (n=6), two for EOPE and LOPE (n=4), and one study provided outcomes for each of the three trimesters (n=3).

Most studies were conducted in Europe (47.4%), followed by Africa (26.3%), Asia (15.8%), and North America (10.5%). Regarding study design, case-control studies were the most frequent (42.1%), while nested case-control and prospective cohorts accounted for 15.8% and 21.1%, respectively. Sampling was most performed during the third trimester (36.8%), followed by the first (31.6%) and second (26.3%), with one study spanning all trimesters. Enzyme-Linked Immunosorbent Assay (ELISA) was the predominant quantification method (68.4%), whereas Luminescent Immunoassay (LIA) was applied in 31.6% of the studies (**Table 1**).

**Table 1:** Characteristics of the included studies in the meta-analysis.

Author	Year	Demographic area	Study design	Trimester at sampling	Methods	Control N Size	PE N Size
Younis et al.	2025	Egypt	Case-control	2nd	ELISA*	20	50
Sun et al.	2023	China	Nested case-control	2nd	ELISA*	96	96
Ersbøll et al.	2021	Denmark	Cohorts	3rd	LIA**	28	28
Marek et al.	2021	Poland	Case-control	1st, 2nd, 3rd	ELISA*	37	21
Neuman et al.	2020	Netherland	Cohorts	3rd	LIA**	250	134
Deepnarain et al.	2020	South Africa	Case-control	3rd	ELISA*	16	16
Jadli et al.	2019	India	Case-control	1st	ELISA*	105	41
Aboelmagd et al.	2018	Turkey	Prospective cross-sectional	1st	ELISA*	63	17
Yeşil et al.	2017	Turkey	Cross-sectional	2nd	ELISA*	67	21
Beljan et al.	2017	United States	Prospective cohort	1st	ELISA*	29	3
Jadli et al.	2017	India	Nested case-control	1st	ELISA*	112	33
Birdir et al.	2015	Germany	Case-control	1st	LIA**	100	35
Tuten et al.	2015	Turkey	Case-control	3rd	ELISA*	40	40
Akinlade et al.	2015	Nigeria	Prospective cohort	3rd	ELISA*	30	60
Santillan et al.	2014	United States	Prospective cohort	3rd	ELISA*	54	50
Wellmann et al.	2014	Switzerland	Prospective cross-sectional	2nd	LIA**	120	27
Yeung et al.	2014	United States	Nested case-control	1st	LIA**	136	169
Foda et al.	2012	Egypt	Prospective cohort	3rd	ELISA*	15	15
Sugulle et al.	2012	Norway	Case-control	3rd	LIA**	71	105
Zulfikaroglu et al.	2011	Turkey	Cross-sectional	2nd	ELISA*	32	64

*ELISA, Enzyme-Linked Immunosorbent Assay.

**LIA, Luminescent Immunoassay.

### 3.3 The concentration of CPP varies in PE and is independent of the region

**Figure 2:**
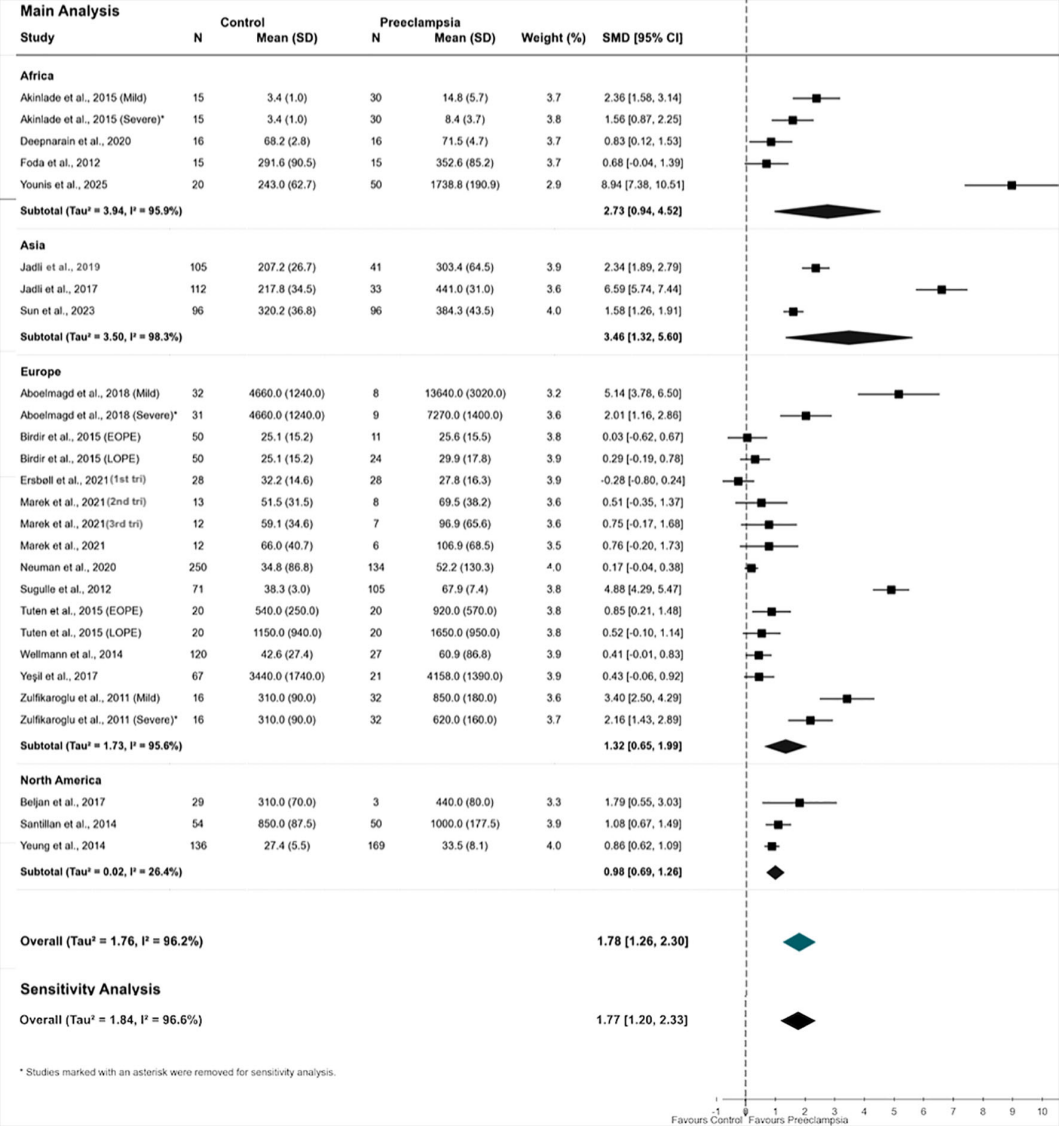
Combined forest plot of the association between CPP levels and PE. The plot displays the Main Analysis (top) and a Sensitivity Analysis (bottom). Studies are grouped by continent. Black squares represent the SMD of individual studies, with the size proportional to their weight. Black diamonds represent the pooled SMD for each continental subgroup. The coloured diamond represents the overall pooled SMD in the Main Analysis. Studies marked with an asterisk (*) were removed for the Sensitivity Analysis, whose results are shown in the bottom diamond.

The results of the meta-analysis are presented in a comprehensive forest plot that combines the main analysis with a sensitivity analysis (**Figure 2**).

A large association was observed between elevated CPP levels and PE and the weight of each study in the analysis ranged from 2.9% to 4.0%. At the overall level, the random-effects model demonstrated a statistically significant association between CPP and PE, with SMD = 1.78 [1.26 - 2.30], alongside a high heterogeneity (Tau² = 1.76; I² = 96.2%). When analysing studies individually, smaller but significant SMD values were observed for Deepnarain et al., 2021 (43) (SMD = 0.83 [0.12 - 1.53]) and Tuten et al., 2017 (SMD = 0.85 [0.21 - 1.48]) for EOPE samples.

In contrast, the largest effects were reported in Younis et al. (44) (SMD = 8.94 [7.38 - 10.51]) and 45 (SMD = 6.59 [5.74 - 7.44]). After removing the severe PE samples by Aboelmagd et al. (25)Zulfikaroglu et al. (46); and Akinlade et al. (47), marked with an asterisk in the main analysis, the model showed a reduced but more precise effect size, SMD = 1.77 [1.20 - 2.33], however, no difference was seen in the high heterogeneity (Tau² = 1.84; I² = 96.6%). Subgroup analysis by region revealed that the association between CPP and PE was statistically significant across all continents. The strongest effects were observed in Africa (SMD = 2.73 [0.94 - 4.52]) and Asia (SMD = 3.46 [1.32 - 5.60) with great influence by Younis et al., 2025 (44), followed by Europe (SMD = 1.32 [0.65, 1.99]) and North America (SMD = 0.98 [0.69 - 1.26]).

### 3.4 Potential risk of bias in the meta-analysis

**Figure 3:**
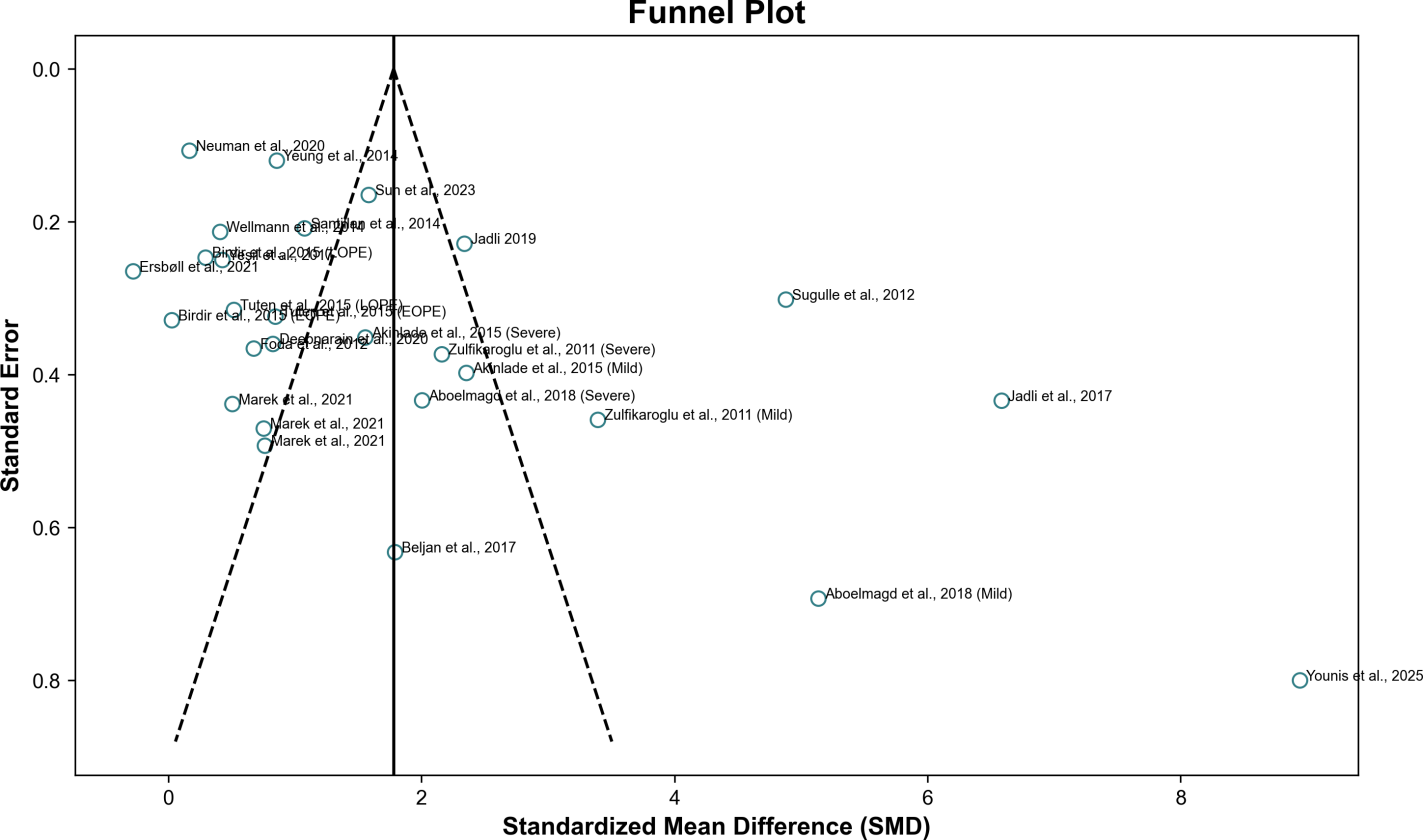
Funnel plot for the assessment of publication bias. The plot maps the effect size (SMD) from each study against its standard error. The vertical line represents the pooled effect size from the main analysis. The dashed lines indicate the 95% confidence interval. The observed asymmetry suggests a potential for publication bias.

To investigate the potential for publication bias, which could influence the meta-analytic findings, a funnel plot analysis was conducted (**Figure 3**). The plot, which maps the effect size of each study against its precision, displayed a notable asymmetry. The scarcity of smaller studies showing non-significant or negative effects (bottom-left quadrant) suggests potential publication bias or small-study effects, likely exacerbated by the high level of heterogeneity between studies.

The complete risk of bias for each included study as well as the summary of the findings were evaluated using the Newcastle-Ottawa Scale score and the GRADE framework for quality of evidence, both provided respectively in **Supplementary Tables 3**, **4**.

### 3.5 Clustering of the control or PE groups based on CPP concentration

**Figure 4:**
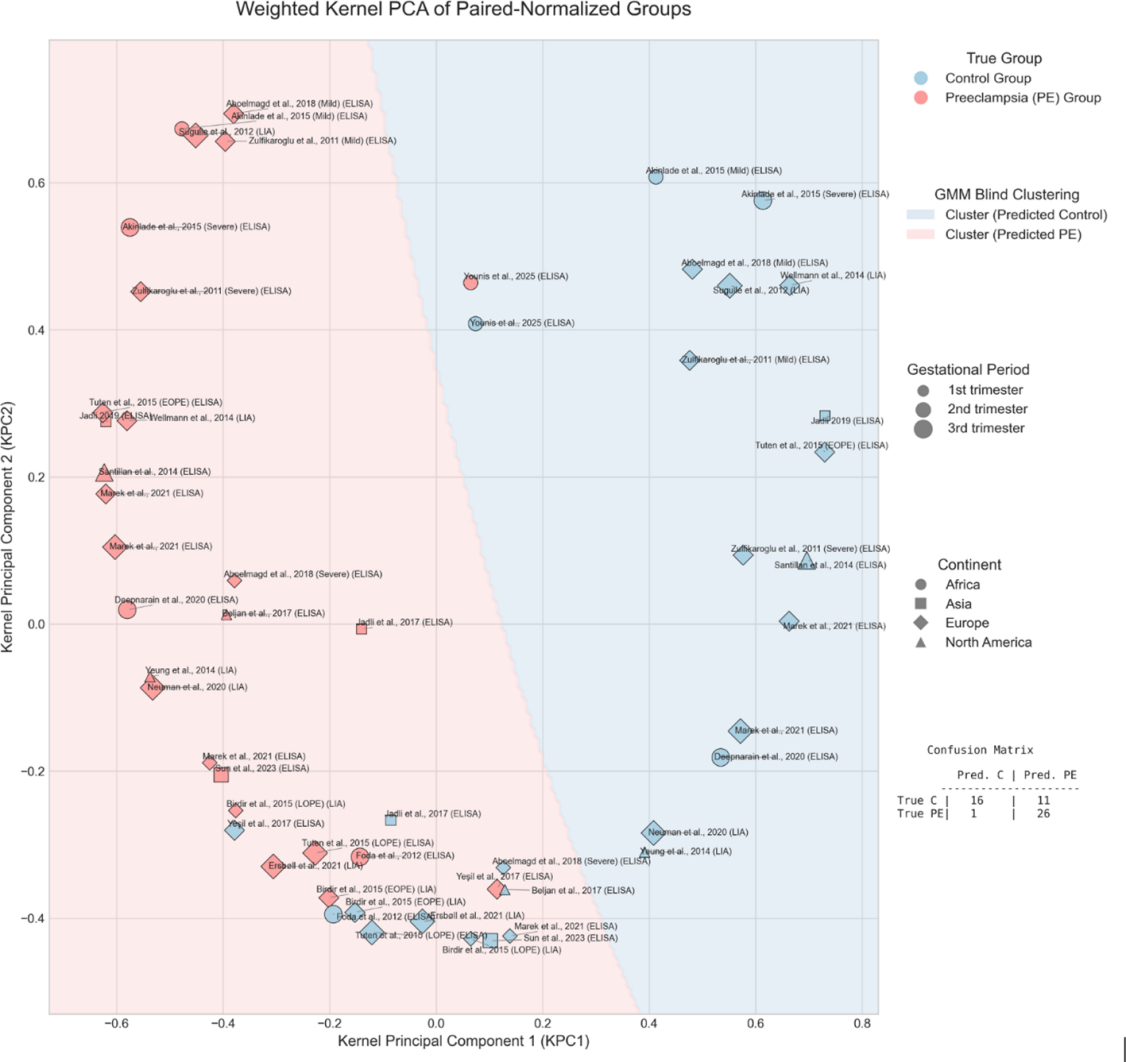
Separation of study groups by Kernel PCA. The first two principal components were plotted to visualize the discrimination between control (blue circles) and PE (red circles) samples according to their CPP profiles. The light blue and light red shaded areas represent the predicted classification boundaries for the two groups. The analysis reveals a distinct clustering of PE samples, while the healthy normotensive pregnancies are diffuse, with a subset of samples overlapping into the predicted PE region. The confusion matrix demonstrates its predictions.

To investigate whether CPP concentrations could naturally discriminate between study groups, we applied the GMM clustering algorithm as an exploratory tool. It is important to emphasize that this analysis utilized study-level aggregated means rather than individual patient data; therefore, the resulting performance metrics reflect the consistency of study findings rather than clinical diagnostic accuracy. The GMM algorithm separated most cases into two clusters that largely aligned with the original study classifications: one cluster was predominantly composed of PE cohort means, while the other consisted mainly of normotensive pregnancies (**Figure 4**).

The confusion matrix, based on the GMM’s blind clustering of these aggregated means, identified eleven control cohorts misclassified within the PE-prevalent region and one PE cohort misclassified as a control. According to these study-level means, the model showed a sensitivity of 96.3% and a specificity of 59.3%. The low specificity observed in this study-level clustering highlights a significant overlap between the characteristics of certain healthy pregnancy cohorts and PE cohorts. These findings suggest that while elevated CPP is a frequent feature of the PE cohorts reported in literature (e.g., 41, 48), the high variance in healthy cohorts limits the use of CPP as a standalone discriminatory marker at this level of aggregation.

### 3.6 PE and AVP-related polymorphisms

Primary research has linked the AVP system to PE through six specific SNPs. One key variant, rs3729965 (Chr 20), affects the promoter region of the gene responsible for AVP, CPP, and the carrier protein Neurophysin II. Erfanian et al. (49) demonstrated this SNP association with PE in Iranian patients (p = 0.028). While this variant resides on chromosome 20, the five remaining SNPs associated with the disorder are found on the *ERAP2* gene on chromosome 5. These SNP frequencies obtained from the literature for the variant alleles are detailed in **Table 2**.

**Table 2:** Characterisation of main SNPs associated with PE.

SNP	Gene function	Variant	Incidence inside/odds ratio	Population and reference
rs3729965 *AVP* Chr 20:3084901	Promoter region	A>G/A>T Highest population MAF: 0.38	p = 0.028	Iran. Erfanian, S., et al. (49) Pregnancy Hypertension
rs2549796 *ERAP2* Chr 5:96909639	Exon (Synonymous Variant)	C>A/C>G/C>T Highest population MAF: 0.49	p = 0.0109 OR = 1.96	Northeast region of Brazil, Ferreira, L.C., et al. (50) Scientific Reports Northeast region of Brazil, Ferreira, L.C., et al. (50) Scientific Reports Northeast region of Brazil, Ferreira, L.C., et al. (50) Scientific Reports
rs2927609 *ERAP2* Chr 5:96916375	Intronic SNP (Popa OM, et al., 2016).	G>A/G>C Highest population MAF: 0.50	p = 0.0109 OR = 1.96	
rs11135484 *ERAP2* Chr 5:96886185	Missense in miR binding sites of RAAS*-related genes.	A>G Highest population MAF: 0.50	p = 0.0109 OR = 1.96	
rs2549782 *ERAP2* Chr 5:96895296	Missense possibly damages the 3-D ERAP2 protein structure in the HEXXH(X)18E motif**.	A>C Highest population MAF: 0.50	p = 0.018	Australia. Johnson MP, et al. (51) Hum Genet
rs17408150 *ERAP2* Chr 5:96903554	Missense possibly damages the ERAP2 protein function.	T>A Highest population MAF: 0.13	p = 0.039	Norway. Johnson MP, et al. (51) Hum Genet

*RAAS, Renin Angiotensin Aldosterone System.

**HEXXH(X), zinc-dependent metalloproteases with active site motif His-Glu-x-x-His (HExxH), zincins are a broad group of proteins involved in many metabolic and regulatory functions and found in all forms of life. The human genome contains more than 100 genes that code for proteins with known zinc-like domains. MAF. Major Allele Frequency (normal).

The rs2549782 missense mutation, involving an ancestral allele frequency of 0.50, was linked to PE in Australia (p = 0.018) due to predicted structural disruption of the HEXXH(X)18E motif. In Norway, the rs17408150 variant also showed significant association (p = 0.039). Research in northeastern Brazil by Ferreira et al. (50), a region where PE incidence (8%) is significantly higher than in the southeast (0.2%), highlighted three further variants, including rs11135484, which affects miRNA binding in RAAS-related genes.

The observed variation in the prevalence of these genetic markers across different global populations suggests a hypothesis that regional differences in PE risk may be influenced by population-specific genetic architectures though further quantitative synthesis is required to confirm any regional link. Besides that, current frequencies of ancestral and variant alleles, obtained from the Ensembl repository (https://www.ensembl.org), remain limited and population-specific (**Table 3**).

**Table 3:** Description of the incidence of SNP stratified by different regions.

SNP	rs3729965 *AVP* Chr 20:3084901	rs2549796 *ERAP2* Chr 5:96909639	rs2927609 *ERAP2* Chr 5:96916375	rs11135484 *ERAP2* Chr 5:96886185	rs2549782 *ERAP2* Chr 5:96895296	rs17408150 *ERAP2* Chr 5:96903554
Globally	0.12	0.5	0.34	0.55	0.45	0.02
AFR	0.01	0.44	0.32	0.59	0.4	0
AMR	0.13	0.57	0.31	0.59	0.42	0.05
EAS	0.07	0.53	0.34	0.52	0.47	NR
EUR	0.27	0.52	0.4	0.53	0.48	0.05
SAS	0.16	0.51	0.31	0.54	0.47	0.02

SNPs, Single Nucleotide Polymorphism; AFR, Africa; AMR, America; EAS, East Asia; EUR, Europe; SAS, South Asia; NR, Not reported.

For instance, the ancestral allele of rs3729965 SNP is nearly fixed in African (AFR) populations at 99%, whereas the variant allele is more frequent in European (EUR) populations (27%). Other markers exhibit more balanced global distributions. The rs2549796 SNP shows an approximate 50/50 split globally, with minor regional fluctuations: the ancestral allele is slightly more prevalent in African populations (56%), while the variant allele is more frequent in American (AMR) populations (57%).

Similarly, the ancestral allele of rs2927609 maintains a consistent global frequency of 66%. Both rs11135484 and rs2549782 show near-equal global distributions, with the latter’s ancestral allele at 55%. In contrast, rs17408150 appears to be the most conserved variant, with the ancestral allele reaching 98% globally and 100% in African and East Asian (EAS) populations. While these geographic trends in SNP frequencies are noteworthy, they currently serve as a foundation for future hypothesis testing regarding regional CPP variations rather than a definitive explanation for clinical outcomes.

## 4 Discussions

From early gestation onward, the HNS undergoes significant adaptations. Accordingly, our findings demonstrate that women with PE consistently present higher standardized CPP concentrations compared to those with normotensive pregnancies, an association that remains consistent regardless of geographic region. These results suggest that increased AVP secretion is a secondary physiological response associated with the clinical progression of PE, reflecting compensatory reactions to immunological, metabolic, and vascular disturbances. Methodologically, a primary strength of this work is its expansion upon the foundational meta-analysis by Bellos et al. (52). By incorporating four recent studies, this analysis provides a more current and comprehensive synthesis of the evidence.

Correlation strength varied across geographic regions, being notably higher in African and Asian cohorts compared to European and American cohorts. This variation suggests a potential role for gene-environment interactions, though current evidence is insufficient to establish a causal link. A primary limitation of current research is the significant geographic bias, with a profound lack of data from underrepresented populations in Africa, Latin America, and Oceania. This disparity renders conclusions on genetic associations less robust. Therefore, future research must prioritize inclusive, global cohorts and integrate advanced methodologies such as genome-wide association studies (GWAS) and epidemiologic genetics to fully elucidate the complex interplay of factors driving the development of PE.

The included observational studies carry a moderate risk of bias, as reflected in the funnel plot’s asymmetry. However, sensitivity analyses confirmed the robustness of the overall effect size, even after the exclusion of samples from patients with severe PE (25, 44, 46). According to the inconsistency indexes (I2), the meta-analysis results indicate that the extreme variability between studies is likely explained by methodological factors rather than chance. Our findings demonstrate variation in the effect sizes of the included trials, a pattern consistent with the comparison reported by Bello et al. This high heterogeneity suggests that reported differences in CPP levels are influenced by diverse clinical protocols, population characteristics, and technical disparities across the primary research.

To further investigate these sources of variance, we performed meta-regression analyses to assess the impact of gestational trimester and assay type (ELISA vs. LIA). The results confirmed that neither assay type (p=0.425) nor trimester (p=0.659) significantly influenced the pooled effect size. This suggests that the association between elevated CPP and PE is relatively robust across different stages of pregnancy and laboratory methodologies. Furthermore, we accounted for inter-assay differences—such as varying detection limits and the lack of universal calibration between commercial kits—by standardizing all data into common units (pg/mL) and utilizing the Standardized Mean Difference (SMD) method. This approach effectively neutralizes the impact of differing calibration scales across platforms, allowing for a more reliable comparison of relative biomarker elevation.

To investigate the influence of disease severity on the CPP concentration, a sensitivity analysis was performed by excluding samples from recruited patients with severe PE (25, 44, 46). This step ensured a more robust estimation of the standardized mean difference of CPP concentration on PE outcome while the effect size remained strong, demonstrating the robustness of the primary finding and reinforcing that methodological differences are the primary drivers of the variation in the results. This suggests that the observed association is not driven solely by cases of severe disease and likely reflects a genuine physiological link present across the PE spectrum. The extreme effect sizes observed in certain cohorts (e.g., SMD >6 in 44 and 45) were manually verified; these values are mathematically consistent with the remarkably small standard deviations reported in those specific populations. Ultimately, the stability of the effect size following the removal of severe PE cases reinforces that the observed association is not driven solely by advanced disease stages but likely reflects a physiological link to PE.

The applied clustering strategy using a Gaussian Mixture Model (GMM) approach, following paired normalization and kernel Principal Component Analysis (kPCA), identified 26 of 27 PE cohorts based on mean CPP concentrations. While this suggests that CPP levels may align with broader PE pathophysiology, it is critical to note that this analysis utilized study-level aggregated means rather than individual patient data. Consequently, the resulting clustering performance, including accuracy and sensitivity, serves as an exploratory visualization rather than a clinically meaningful diagnostic metric. The high overlap with healthy pregnancy cohorts resulted in low specificity, which limits the immediate clinical utility of this specific model. These findings likely reflect underlying data heterogeneity rather than methodological failure. For instance, the misclassified healthy cohorts may represent a subset of pregnancies undergoing significant physiological stress that elevates CPP without the clinical manifestation of hypertension. This highlights the potential of machine learning to assist in statistical research (53), though its application here remains hypothesis-generating.

The observed heterogeneity in CPP levels may also be influenced by factors such as ethnic background, geographic location, or the specific PE phenotype (e.g., early-onset vs. late-onset). In this context, CPP may serve a dual role: it functions as a surrogate biomarker reflecting systemic neuroendocrine stress and may also represent a mechanistic mediator through the dysregulation of the Hypothalamic-Neurohypophysial System (HNS). Persistent AVP elevation, as indicated by elevated CPP, has been associated with the endothelial imbalance seen in both EOPE and LOPE (54). However, whether AVP axis dysregulation is a primary driver or a secondary response to placental dysfunction requires further experimental validation.

While the HNS adapts to ensure adequate placental perfusion, it clearly malfunctions in PE, contributing to the disease’s multifactorial pathogenesis. Yoshihara et al. (55) reinforced that dysregulated AVP metabolism by placental enzymes leads to hormonal imbalances. In murine models, chronic AVP exposure during gestation is sufficient to induce cardiovascular and renal phenotypes mirroring human PE, while also driving characteristic immunological alterations (56). This hormonal disruption is further associated with the dysregulation of placental genes related to transport and energy metabolism (57). Beyond its predictive capacity, elevated CPP is linked to PE pathophysiology, demonstrating associations with abnormal uterine artery Doppler findings (46), elevated blood pressure and creatinine levels (58), and adverse neonatal outcomes (47).

We examined the population-specific frequencies of SNPs in the *AVP* and *ERAP2* genes as a candidate mechanism for AVP axis dysregulation. In theory, these polymorphisms could influence hormone transcription or impair the enzymatic clearance of AVP, potentially leading to prolonged bioavailability and maladaptive vasoconstriction. However, as these cited studies are few and population-specific, these genetic patterns should be interpreted as hypothesis-generating. The varying frequencies of these variants across global populations do not yet provide a definitive explanation for regional PE disparities but do underscore the need for further quantitative synthesis. These discrepancies emphasize that CPP testing must be considered within a precise clinical context. Given that PE is multifactorial, CPP may be most effective not as a stand-alone tool, but as a component of multi-marker panels paired with factors such as the sFlt-1/PlGF ratio or BMI (45, 54) to refine comprehensive risk assessments.

## 5 Conclusions

Advancing the understanding of biomarkers like CPP is essential for addressing the physiological complexities of preeclampsia. The findings of this meta-analysis reinforce that elevated peripheral CPP levels in pregnancy are associated with PE. Rather than indicating primary AVP secretory dysfunction, this association likely reflects a compensatory physiological mechanism. Instead, /these elevated levels may reflect a compensatory physiological response to the hemodynamic and metabolic stresses associated with PE, including variations in vascular tone and plasma osmolality.

While the clustering analysis demonstrates that CPP levels often distinguish PE cohorts from normotensive pregnancies, the overlap between groups suggests that CPP may be most effective as a component of multi-marker risk assessment strategies rather than a standalone tool. Subgroup analyses revealed stronger associations in African and Asian cohorts, highlighting the importance of considering geographic and ethnic diversity in future research. The reasons for these regional disparities remain a subject for future hypothesis testing, and current data do not support definitive conclusions regarding specific evolutionary or causal genetic adaptations. Future research should focus on individual patient data meta-analyses and machine learning models that can better account for clinical and methodological heterogeneity to refine the predictive value of the AVP axis in PE.

## Data Availability

The original contributions presented in the study are included in the article/**Supplementary Material**. Further inquiries can be directed to the corresponding author.
