# Supplementary material for: The hypothalamic-neurohypophyseal system in preeclampsia: a systematic review with a subgroup meta-analysis of copeptin levels worldwide
Source: Front Endocrinol (Lausanne). 2026 Jun 10;17:1796685. doi: 10.3389/fendo.2026.1796685 (PMC13311781; doi:10.3389/fendo.2026.1796685)
Supplement: Supplementary file 1 [file DataSheet1.pdf]

SUPPLEMENTAL MATERIAL

Supplementary Table 1. Characteristics of the thirty-nine primary studies included in the systematic review detailing the author, year of publication and title for each study.

Authors	Year	Title
Younis et al.	2025	Genetic Factors in Preeclamptic Egyptian Women: Relation with Arginine Vasopressin; A Case-Control Study
Vignato et al.	2023	Selective Serotonin Reuptake Inhibitor Use in Pregnancy and Protective Mechanisms in Preeclampsia
Sun et al.	2023	The relationship between arginine vasopressin gene polymorphisms and plasma copeptin and hypertensive disorders of pregnancy: a nested case-control study
Gray et al.	2023	Cellular Immunotherapy in Mice Prevents Maternal Hypertension and Restores Anti-Inflammatory Cytokine Balance in Maternal and Foetal Tissues
Reddy et al.	2023	Hesperidin improves physiological outcomes in an arginine vasopressin rat model of pre-eclampsia
Shir-Jing Ho et al.	2023	Proteomic studies of human placentas reveal partnerships associated with preeclampsia, diabetes, gravidity, and labor
Gumusoglu et al.	2022	Effects of Maternal Hypertension on Cord Blood Arginine Vasopressin Receptor Expression
Ramdin et al.	2022	Physiological characterization of an arginine vasopressin rat model of preeclampsia.
Gumusoglu et al.	2021	Altered offspring neurodevelopment in an arginine vasopressin preeclampsia model
Ersbøll et al.	2021	Biomarkers and Their Relation to Cardiac Function Late After Peripartum Cardiomyopathy
Marek et al.	2021	Copeptin in Patients with Pregnancy-Induced Hypertension
Ferreira et al.	2021	Genetic association of ERAP1 and ERAP2 with eclampsia and preeclampsia in northeastern Brazilian women
Neuman et al.	2020	Copeptin and mid-regional pro-atrial natriuretic peptide in women with suspected or confirmed pre-eclampsia: comparison with sFlt-1/PlGF ratio.
Deepnarain et al.	2020	Are the Circulating Levels of Copeptin and Fibronectin Dysregulated in Preeclamptic South African Black Women?
Erfanian et al.	2019	Association of arginine vasopressin (AVP) promoter polymorphisms with preeclampsia
Jadli et al.	2019	Prediction of preeclampsia using combination of biomarkers at 18–23 weeks of gestation: A nested case-control study
Gao et al.	2019	Hyper-methylation of AVPR1A and PKCB gene associated with insensitivity to arginine vasopressin in human pre-eclamptic placental vasculature
Fan et al.	2019	DNA methylation-reprogrammed oxytocin receptor underlies insensitivity to oxytocin in pre-eclamptic placental vasculature
Sandgren et al.	2018	Arginine vasopressin infusion is sufficient to model clinical features of preeclampsia in mice
Scroggins et al.	2018	Elevated vasopressin in pregnant mice induces T-helper subset alterations consistent with human preeclampsia
Aboelmagd et al.	2018	Maternal Serum Copeptin for Early Prediction of Preeclampsia
Yeşil et al.	2017	Identification of patients at risk for preeclampsia with the use of uterine artery doppler velocimetry and copeptin
Beljan et al.	2017	Can first trimester placental biomarkers copeptin and PP13 predict preeclampsia in advanced age nulliparous women?
Jadli et al.	2017	Combination of copeptin, placental growth factor and total annexin V microparticles for prediction of preeclampsia at 10-14 weeks of gestation.
Birdir et al.	2015	Maternal serum copeptin, MR-proANP and procalcitonin levels at 11-13 weeks gestation in the prediction of preeclampsia.
Tuten et al.	2015	Maternal serum copeptin concentrations in early- and late-onset pre-eclampsia
Akinlade et al.	2015	Serum copeptin and pregnancy outcome in preeclampsia.
Wellmann et al.	2014	Cardiovascular biomarkers in preeclampsia at triage.
Yeung et al.	2014	Increased Levels of Copeptin Before Clinical Diagnosis of Preeclampsia
Santillan et al.	2014	Vasopressin in Preeclampsia: A Novel Very-Early Human Pregnancy Biomarker and Clinically-Relevant Mouse Model
Foda et al.	2012	Maternal and neonatal copeptin levels at cesarean section and vaginal delivery.
Sugulle et al.	2012	Cardiovascular risk markers in pregnancies complicated by diabetes mellitus or preeclampsia
Zulfikaroglu et al.	2011	Circulating levels of copeptin, a novel biomarker in pre-eclampsia.
Johnson et al.	2009	The ERAP2 gene is associated with preeclampsia in Australian and Norwegian populations
Ong et al.	2002	Functional characteristics of chorionic plate placental arteries from normal pregnant women and women with pre-eclampsia.
Gordge et al.	1994	Serum Vasopressinase and Platelet Responses to Arginine Vasopressin in Normal Pregnancy, Pregnancy-induced Hypertension and Pre-eclampsia.

Van der Post et al.	1993	Preeclampsia is not associated with altered platelet vasopressin binding and cytosolic Ca++ concentration
Allen et al.	1989	Effect of endogenous vasoconstrictors on maternal intramyometrial and foetal stem villous arteries in pre-eclampsia
Pedersen et al.	1985	The osmoregulatory system and the renin-angiotensin-aldosterone system in pre-eclampsia and normotensive pregnancy

Supplementary Table 2. The reasons for each author's exclusion from the meta-analysis.

Author	Year	Title	Excluded from the Meta-analysis
Vignato et al.	2023	Longitudinal biobehavioral and omics pathways to preterm birth: the Early Stress and the Psychobiome Study	Lack of Copeptin means
Gray et al.	2023	The role of placental growth factor-like proteins in the pathophysiology of preeclampsia	Interventional study
Reddy et al.	2023	Exploring the role of neurohypophyseal hormones in maternal-fetal health: a comprehensive review	Narrative review
Ho et al.	2023	Vasopressin and copeptin in pregnancy and preeclampsia: a review of current evidence and future directions	Missing primary Copeptin data
Gumusoglu et al.	2022	Copeptin and placental development: effects on human umbilical vein endothelial cells	In vitro study
Ramdin et al.	2022	Effects of a simplified management protocol on outcomes in preeclampsia with severe features	Interventional study
Ferreira et al.	2021	Copeptin as a potential biomarker for preeclampsia: a systematic evaluation of current evidence	Insufficient quantitative data
Gumusoglu et al.	2021	Efficacy of copeptin as a diagnostic tool in preeclampsia: a randomized clinical trial protocol	Clinical trial protocol
Gao et al.	2019	The effect of magnesium sulfate on copeptin levels in patients with severe preeclampsia	Interventional study
Fan et al.	2019	Transcriptomic analysis of the neuro-placental axis in a murine model of preeclampsia	Missing Copeptin protein levels
Sandgren et al.	2018	Vasopressin in preeclampsia: from mice to humans	Animal model/Translational trial
Scroggins et al.	2018	Elevated copeptin levels in early pregnancy are predictive of preeclampsia	Interventional study
Nizyaeva et al.	2017	Expression of vasopressin receptors in placenta of women with preeclampsia	Closed access
Johnson et al.	2009	Genetic variations in the vasopressin system and the risk of preeclampsia: a case-control study	Genetic analysis (SNP focus)
Ong et al.	2002	The use of copeptin in the prediction of preeclampsia: a prospective controlled trial	Incompatible sampling protocol
Gordge et al.	1994	Arginine vasopressin and platelet activation in pre-eclampsia	Incompatible matrix (Platelet AVP)
Van der Post et al.	1993	The role of the neurohypophysis in pregnancy-induced hypertension	Incompatible matrix (Whole blood)
Allen et al.	1989	Arginine vasopressin levels in hypertensive pregnancy	Interventional study
Pedersen et al.	1985	Vasopressin and oxytocin in the mother and fetus during labor and delivery	Incompatible matrix (Cord blood)

Supplementary Table 3. Newcastle-Ottawa Scale score of the cohort and case-control studies included in the meta-analysis.

Author	Year	Registries	Selection	Comparability	Exposure/ outcome	Total
Younis et al.	2025	Nested case-control	3	1	3	7
Sun et al.	2023	Nested case-control	3	1	3	7
Ersbøll et al.	2021	Prospective cohort	2	1	3	6
Marek et al.	2021	Case-control	3	2	3	8
Neuman et al.	2020	Prospective cohort	3	2	3	8
Deepnarain et al.	2020	Case-control	3	2	3	8
Jadli et al.	2019	Nested case-control	3	2	3	8
Aboelmagd et al.	2018	Prospective cohort	3	2	3	8
Yeşil et al.	2017	Cross-sectional	2	1	3	6
Beljan et al.	2017	Prospective cohort	2	2	3	7
Jadli et al.	2017	Nested case-control	3	2	3	8
Birdir et al.	2015	Nested case-control	3	1	3	7
Tuten et al.	2015	Case-control	3	2	3	8
Akinlade et al.	2015	Prospective cohort	3	2	3	8
Santillan et al.	2014	Prospective cohort	2	1	3	6
Wellmann et al.	2014	Cross-sectional	3	2	3	8
Yeung et al.	2014	Prospective cohort	3	2	3	8
Foda et al.	2012	Cross-sectional	3	2	3	8
Sugulle et al.	2012	Cross-sectional	3	2	3	8
Zulfikaroglu et al.	2011	Cross-sectional	3	1	3	7

Supplementary Table 4. Summary of the findings and quality of evidence of the comparison of cases included in the meta-analysis using the GRADE platform.

Comparison	Healthy	PE	SMD [95% CI]	Quality
PE vs. Healthy	1421	1025	1.78 [1.26, 2.30]	⊕⊕⊕○ Moderate
